# Prescribing for personality disorder: qualitative study of interviews with general and forensic consultant psychiatrists

**DOI:** 10.1192/pb.bp.113.044081

**Published:** 2014-06

**Authors:** Lawrence Martean, Chris Evans

**Affiliations:** 1 Nottinghamshire Healthcare NHS Trust, UK

## Abstract

**Aims and method** To explore experiences of psychiatrists considering medication for patients with personality disorder by analysis of transcribed, semi-structured interviews with consultants.

**Results** Themes show important relational processes in which not prescribing is expected to be experienced as uncaring rejection, and psychiatrists felt helpless and inadequate as doctors when unable to relieve symptoms by prescribing. Discontinuity in doctor-patient relationships compounds these problems.

**Clinical implications** Problems arise from: (a) the psychopathology creating powerful relational effects in consultation; (b) the lack of effective treatments, both actual and secondary to under-resourcing and neglect of non-pharmaceutical interventions; and (c) the professionally constructed role of psychiatrists prioritising healing and cure through provision of technological interventions for specific diagnoses. There is a need for more treatments and services for patients with personality disorder; more support and training for psychiatrists in the relational complexities of prescribing; and a rethink of the trend for psychiatrists to be seen primarily as prescribers.

Since the 1950s the number of psychotropic medications available has increased.^1^ Drug treatments showed utility for psychoses^2^ and depression,^3^ although arguably the benefit has been overvalued.^4-6^ By contrast, there is little evidence of utility for people with personality disorder. Current guidelines give limited support for medication^7-9^ and no clear justification for long-term medication.^10,11^ Consequently, psychiatrists encounter patients with personality disorder with persisting symptoms, disability and unmet needs.^12^ In addition, drug treatments have to be considered repeatedly and despite guidance many patients are treated largely or entirely with medication. There is evidence of high rates of polypharmacy for patients in secondary care mental health services^13-15^ and evidence that prescribing for patients with personality disorder may vary according to the severity of symptoms, the risks of offending behaviours and with service contexts.^16^

The UK National Institute for Health and Care Excellence (NICE) guidelines on borderline and antisocial personality disorders^17,18^ provide aspirational frameworks intended to standardise practice. Those guidelines pay lip service to relational issues in consultations but cite little evidence for this. Exploration of doctors’ decision-making is increasing^19^ and research suggests that doctors frequently deviate from clinical guidelines for many reasons, including sound clinical appraisal.^20-23^ However, evidence about how prescribing decisions are made by psychiatrists is very limited by comparison with the volume of randomised controlled trial (RCT) research evaluating medication.

This study sought to explore consultants’ experiences of and views on prescribing for patients with personality disorder, applying a thematic analysis to find the themes that were important to participants and which emerged consistently.

## Method

### Participants

The study was approved by a National Health Service (NHS) research ethics committee. Interviews with 11 consultant psychiatrists were conducted in 2010. The consultants worked in community mental health teams, crisis and home treatment teams, assertive outreach, rehabilitation services, acute in-patient services and forensic services (predominantly high secure).

### Procedures

All interviews were conducted by L.M. using a semi-structured schedule and interviews lasted up to 1 h. Questions were asked to elicit factors which influenced the participants’ experiences during consultations about prescribing for patients with personality disorder (e.g. what aided decision-making, what did not help and any factors which they experienced as barriers to effective decision-making) and were asked to reflect on the emotional aspects of these experiences and their views on how their feelings effected their communications, actions or decisions. The interview focused on how the experience differs from prescribing for Axis I disorders.

### Data analysis

Interviews were transcribed and analysed by L.M. using thematic analysis.^24,25^ Analysis started with coding of initial themes for manifest data which occurred commonly and writing memos about latent content for possible emergent themes. The first six interviews were fully analysed to develop a coding frame and subsequent interviews analysed using this. Theme saturation was reached with no new major themes arising from the final transcript. The first six interviews were read in full by C.E. and code development was checked and discussed. Disagreements largely took the form of adding codes rather than any disagreements about use of codes. For the remaining interviews, memos made by L.M. were reviewed to establish whether new themes should be included, and coding that felt debatable was discussed.

## Results

**Fig 1:**
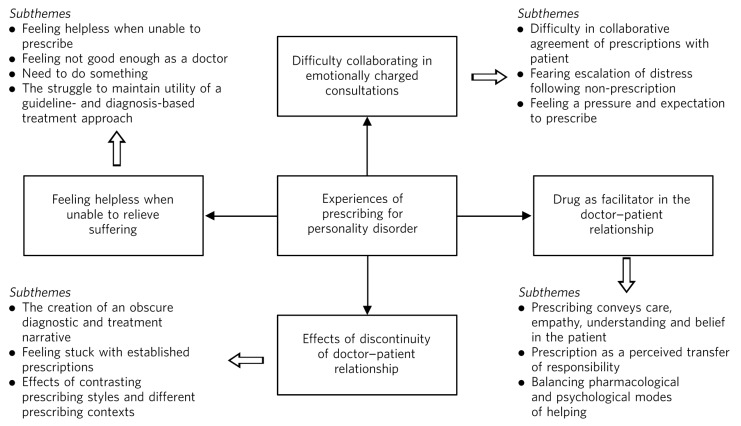
Summary of results.

There were four main themes consistently present across the transcripts, with 13 subthemes (Fig. 1). The results are detailed below by presenting segments of the interview transcripts which are examples of the same units of text used for coding during analysis to produce the final themes and subthemes.

### Difficulty collaborating in emotionally charged consultations

Participants repeatedly described being unable to reach satisfactory agreement with patients regarding prescribing, and most described examples of high levels of distress and conflict in some consultations. The core tension was between making a clinically sound but unpopular decision or agreeing to prescribe to attempt to preserve a positive doctor-patient relationship.

#### Difficulty in collaborative agreement of prescriptions with patient

‘He just would not accept being on no medication. Of course I could just not prescribe, I could just say it doesn’t matter what you say, I am not going to prescribe anything. I just didn’t feel able to do that. It seemed that if he didn’t get that, he wasn’t going to engage’ (P10).

‘It just felt to me that the projection was so strong to the point that the person was telling me that they thought I had the same eyes as their abusive father. It was just getting corrosive really so I asked for that person to be taken over by someone else’ (P6).

#### Fearing escalation of distress following non-prescription

Many psychiatrists described a feeling of dread, anticipating high-emotion responses and conflict about not prescribing.

‘Sometimes they will be really angry or upset if they are not getting what they want, they storm out of your office or start shouting at you or things like that’ (P4).

#### Feeling pressure and an expectation to prescribe

Intense pressure could come from the patient, relatives, colleagues or society, but also from within. Some participants felt more confident than others to resist these pressures and several felt resisting was dependent on the context in which they were practising.

‘They see you as a doctor, they see you as a prescriber. They know you are a psychiatrist. Patients come to see you knowing that you can prescribe’ (P2).

‘It is difficult, you are in a team aren’t you, you feel an obligation to protect your nursing staff but you have got to do what is best haven’t you, and your first responsibility is to your patient and you have got to do what is best for them regardless of [the] kind of other pressures that come’ (P8).

Some described concerns for their own safety, especially in forensic services.

‘If she wants to talk about medication, we get lots of staff who stand very close and I stand very, very far away because she has already kicked me once and I am not making that mistake again’ (P11).

### Feeling helpless when unable to relieve suffering

There were many quotes highlighting helplessness and powerlessness when faced with a patient who is suffering. Pressures included feeling restricted by guidelines, not having the time or the expertise to offer non-pharmacological interventions and having poor access to psychological treatments.

#### Feeling helpless when unable to prescribe

‘There is something really sad when you have got someone sitting in front of you saying please help me, but it is a long-term problem that you can help in the long-term but you can’t do anything there and then. They are so used to being told in prisons particularly, well try this pill, and it hasn’t worked. So that is really difficult - that sense of helplessness at that point’ (P11).

‘What can you offer to them? Well I can refer you to the [psychological therapy] group, but it is a group and you have to be assessed and there is a few months’ waiting list. It doesn’t fit with the need of the patient at the time. There is something about containing the situation here and now, which you think is all there on your shoulders’ (P1).

#### Feeling not good enough as a doctor

‘They are expecting to be given a prescription because they are seeing a doctor. When you tell them “no” then they say, well why did we come here, what was this appointment for, are you doing your job properly, you don’t understand what is going on’ (P4).

‘I think that personally I have found it incredibly difficult because I have felt that I have failed; as a doctor I have failed to alleviate someone’s distress and in many regards maybe I have made it worse’ (P2.)

#### The need to do something

‘On occasions I don’t actually need to do anything. And that is actually quite a hard lesson for a medic. I think you know that you can end a consultation and say, “Well I am sorry you feel like this; hopefully things will change. I am going to see you again in 2 months”. That is actually very hard for medics to do, because what we want is for people to actually be able to say, “Well thank you doc, I feel much better”, and go’ (P2).

‘At one level I am responding to a request for help and often the request comes in a language - I don’t really care what you do, just make things better for me’ (P7).

#### The struggle to maintain utility of a guideline- and diagnosis-based treatment approach

‘I think some clinicians in some places are keener to call something psychosis perhaps when it doesn’t look like that to me, so that they can then prescribe safely... and I can sort of understand that approach from clinicians really that a severely personality disordered individual with impulsivity or whatever might, with a particular type of voice which might be normally thought of as a personality type of voice, might be called psychotic, so that you can then prescribe for it’ (P8).

‘For me, personality disorder is much more symptom led - it is much more about level of disability by symptoms, severity of symptoms and then going with a means to an end, [prescribing] as an adjunct to get them into a psychological treatment so it is not diagnostic led, I don’t think, prescribing in [personality disorder]’ (P5).

‘They often produce very polarised views among staff, and sometimes among doctors as well about, well, what is this: is it schizophrenia, is it borderline personality disorder, how should we be managing this, surely we should be taking them off their medication, sending them away and saying they have responsibility for themselves, etc., etc.? And other staff will say, “No, this person is psychotic, they need our paternalistic medicalised care’” (P3).

### Effects of discontinuity in the doctor-patient relationship

#### The creation of an obscure diagnostic and treatment narrative

‘Patients tend to come in on a huge raft of different medications because they tend to have failed elsewhere, by which time they will have accrued six or seven different kinds of medications. They don’t know which one is helpful, they don’t know if anything is helpful in the least anyway’ (P11).

‘Well I suppose they will be on medication from every group - antidepressant, mood stabiliser, antipsychotic. That is often the case, with not a very clear rationale. If you were not the one who did this prescribing, then it is hard to actually see by retrospectively searching through the notes, what was started at which point and why. Things seem to be just added and I think it reflects that people felt that we just need to do something... well this doesn’t work so I’ll add this, or this doesn’t work so I’ll add another’ (P1).

The psychiatrists working in acute care gave stark warnings about the effects of fragmented patient journeys through services with rapid changes in doctor-patient relationships.

‘When [patient care] gets chopped up and broken into different people making all the decisions I think it leads to quite a bit of anxiety and lack of containment that can put people into crises and lead to inappropriate prescribing [...] We know that consistency is probably the most important thing, and that is something that has been very much lost in our services’ (P3).

#### Feeling stuck with established prescriptions

‘When patients have been misdiagnosed and been given lots of different medications, then to try and gradually work in the idea that this is a longstanding problem ingrained into their personality and maybe we should look at other treatment options. I suppose trying to focus on other treatment options is, umm, is that they don’t really like to be told that. If they have just been told that medication is the only thing for them and you are taking it away, it may feel as though you are taking all help away from them and there are no sort of alternatives’ (P4).

#### Effects of contrasting prescribing styles and different prescribing contexts

‘If that person goes from one to another service, to another psychiatrist then gets very different views, that can make life very hard for the people. They feel very uncontained, they don’t know what’s right, what’s wrong, and they’ll much more often be attracted to the more medical model of their care because of the potential, the ongoing potential for instant rewards’ (P3).

### The drug as a facilitator in the doctor-patient relationship

#### Prescribing conveys care, empathy, understanding and belief in the patient

The participants gave detailed insights into how the act of prescribing is inseparable from the relational processes in consultations with patients with personality disorder.

‘It was very difficult to not give him medication, because he wanted it so much and it seemed to be all linked up with “If you don’t give me medication then you don’t believe that I am ill and so you are rejecting me” [...] I think medication is like the milk, the mother’s milk, and we have to wean him off quite slowly’ (P9).

‘It is just an issue when you are trying to reduce it, because if their view of themself is that they are ill, and they need to be ill to get care and they see a reduction in their medication as a sign that everyone thinks that they are not ill, then I think that causes them panic and can destabilise them’ (P10).

‘Making the doctor feel that they have actually done something, so everyone briefly feels better, although ultimately the patient doesn’t benefit from that. What the patient may have benefitted from in the short term at least is being heard, yeah, and that prescribing might be part of that process I guess. But what I am saying is that if we can work with them consistently, that sense of them needing to be heard by giving them medicine needs to be removed from the equation. We need to hear them, and the way we hear them is by spending long enough with them to not give them medication’ (P2)

#### Prescription as a perceived transfer of responsibility

‘I think by giving people tablets if you are not careful you do remove that sense of responsibility. It is almost like, these tablets aren’t working therefore it is the doctor’s fault that I hit that person’ (P1).

‘On the one hand you are saying you should be responsible for your actions and you have got to work in this way, but on the other hand you are giving them a slightly mixed message which is we’ll put you on these tablets so you have got an illness, you know, that sort of conflict. I think it is much more complicated if you are prescribing and the patient had got a prescription because of the power, the powerful symbolism of that and the fact that self-harming can arise from that [...] It feels different if someone self-harms with something you have given to them, than with something you haven’t given to them [...] I think that can be a very powerful thing between the doctor and the patient if they are potentially going to self-harm with what you are giving them’ (P10).

#### Balancing pharmacological and psychological modes of helping

‘She does become very dissociated and it is difficult for us to have any kind of conversation. If we do have a conversation she often can’t respond to me - it’s me just being with her really and trying to talk to her but then often after the event she can’t remember an awful lot of what I have said to her [...] I can’t negotiate any kind of care plan with her because she is so distracted she can’t really talk. So when people are that kind of distracted and dissociated I think that medication can be useful so you can just start to even think about a plan together’ (P10).

‘My approach to prescribing is that in order to get them into the psychological therapy, get their, reduce their level of arousal, reduce their level of paranoia, reduce their level of kind of emotional dysregulation, I will prescribe some medication to get them into the treatment and then I will start to, as they acquire psychological skills, I will then tail off the medication’ (P5).

## Discussion

These findings support the view that managing medication and achieving collaborative decision-making for patients with personality disorder is complex and challenging. This seems consistent with the ‘mentalisation’ model that highly distressed patients, particularly with borderline pathology, are unable to reflect on their own mental states or those of the doctor,^26^ a capacity probably necessary for negotiation and agreement. The doctor may possess a great desire to help and relieve suffering based on both personal experience and training, and the wish to do something can become a problem when faced with a patient who does not appear to get better.^27^ This impulse to do something, which may be helpful with other patient groups, may provide a disabling complementarity between doctor and patient.

Bateman & Fonagy describe the ‘non-mentalising’ patient who is prone to interpret only physically observable actions as evidence of changes in mental states of others, meaning that prescribing may be felt as the only evidence of genuine care and concern. In mentalisation terminology, the ‘teleological mode’ of relating^28^ may be likened to a phenomenon in which ‘actions speak louder than words’. This may help explain why psychiatrists sometimes use prescribing as a way of communicating empathy and establishing rapport with the patient when there may be mutually high emotional arousal during difficult consultations.

It is clear from these interviews that consultants experience consultations with patients with personality disorder as emotionally charged and that doctors sometimes prescribe to preserve a good doctor-patient relationship.^29^ Clinical guidelines do not adequately take into account the relational aspects of prescribing, leaving doctors struggling to follow prescribing guidance when under great pressure to do something.^30^

This study also highlights the uncertainty that psychiatrists experience when attempting to practice within a diagnosis-based treatment paradigm for the most complex and challenging patients, with sometimes very little apparent utility of diagnosis in informing the actual prescribing decisions. The social and professional construction of the diagnosis of personality disorder may be controversial^31^ but can be difficult to question in the heat of the consultation and may set up a role for the psychiatrist as ‘healer’, endowed with expectations of cure relating to diagnosis not to problems - and there is evidence that problems as much or perhaps more than diagnosis may be crucial to explore for patients with personality disorder.^32-34^ A shift to consider problems and states rather than diagnoses may be congruent with new views of the action of psychotropic drugs as modifiers of general mental states rather than treatments for illness-specific pathology.^35^

The study underlines that ‘non-patient factors’^36^ can be strong determinants of prescription patterns, including: access to psychological treatments and specialist services, prescriber view of medication effectiveness and the type of institution in which the patient is encountered.^37^ The findings also suggest that the lack of continuity of the doctor-patient relationship obscures diagnosis, leads to difficulty in modifying established prescriptions and may predispose to crises resulting in overprescribing. Evidence of high rates of polypharmacy in this group is gathering strength and the phenomenon of overtreatment in medicine in general is increasingly recognised^38,39^ and the many reasons for this need more exploration.

This study sheds light on reasons why prescribing practice for patients with personality disorder may not be helped much by guidelines, whose evidence base is limited to RCTs using highly select groups of patients. Guidelines do not foster a range of empirical exploration of prescribing relationships and complexities as highlighted in this study and this underlines a recent call for a paradigm shift in psychiatry.^40^

### Strengths and limitations of the study

Researcher biases in the interviews and analysis may influence the findings. The usual steps were taken to try to limit these by using a semi-structured interview schedule and by C.E. cross-checking the analysis. Of course, this will only restrict bias, not eliminate it, and we are both psychiatrists with Certificates of Completion of Training in psychotherapy, with a particular interest in interpersonal and affective processes. L.M. has been recently working in a range of in-patient and community mental health service contexts involving consultations with patients with personality disorder when he would make prescribing decisions as part of the role. C.E. has worked largely with patients with personality disorder since about 1990 and believes short-term use of medication has an important role in work with patients with personality disorder, although his own posts over the past 15 years have not involved prescribing. He is involved in a Health Technology Assessment RCT of mood-stabilising medication for patients with personality disorder.

The sample included psychiatrists from a broad range of services and the themes identified were present across the majority of interviews giving confidence they were general, and we felt that thematic saturation was reached. However, the sample was taken from a single trust, although local characteristics would seem fairly generalisable - and the sample was restricted to consultants. Replication in other areas and with extension to non-consultant grades and trainees and with more psychiatrists specialising in work with patients with personality disorder would be valuable. Research into the experiences of other mental health professionals involved with patients with personality disorder should be sought, as triangulation of the doctor-patient relationship in modern multidisciplinary work was a clear and important theme.

### Implications

Our findings support the need for further review of the current approach to providing treatments within NHS services for patients with personality disorder and provide an alternative perspective to the predominantly quantitative research on this topic. The findings show that affective as well as cognitive processes affect consultations, and prescribing decisions may be powerfully influenced by emotional factors in the relationship between doctor and patient. Even experienced consultant psychiatrists can feel ill equipped discussing medication with some patients. The study indicates that psychiatrists attempt to use medication in highly considered ways, for example to enhance engagement and improve ability to access psychological treatments and to manage crises. More research is needed to establish when such tailored prescribing is effective and when and how best to provide these interventions.^41^

Research is also needed into how prescribing decisions and patient care might be improved by training and by specialist supervision and support for psychiatrists in managing the emotional demands of these consultations. More research is also required into the experiences of those in other roles in relation to consultations about prescribing with patients with personality disorder, particularly patients themselves, carers, nurses, trainee psychiatrists and general practitioners.

The study suggests that the current NICE guidance for personality disorder is in need of significant review and updates. In particular, it needs to allow more for the diversity and complexity of the patients presenting and the variety of treatment contexts in which psychiatrists provide this care, but also to foster empirical research into prescribing and discussion of management options so that guidance on this is based on empirical data as much as choices between medications is based on sound double-blind RCT evidence. This is not about criticising the RCT for medication but about recognising that pressures on NICE lead its guideline development processes and particularly its hierarchy of evidence to be skewed far out of line with its true epistemological needs.^42,43^

Although ‘helper helplessness’ may be common and sometimes inevitable in this area, this study highlights the need to improve access to, and provision of, specialist services and effective psychological treatments to avoid psychiatrists feeling there is no alternative but to prescribe. This may support the argument for psychiatrists themselves taking back some of the lost role of providing psychological support and intervention, alongside pharmacological overview, to help them feel less expectation to prescribe and give them tools to provide care at times and situations when drug treatments options are not indicated.
